# Interventions to improve recruitment and retention in clinical trials: a survey and workshop to assess current practice and future priorities

**DOI:** 10.1186/1745-6215-15-399

**Published:** 2014-10-16

**Authors:** Peter Bower, Valerie Brueton, Carrol Gamble, Shaun Treweek, Catrin Tudur Smith, Bridget Young, Paula Williamson

**Affiliations:** National Institute for Health Research School for Primary Care Research, North West Hub for Trials Methodology Research, University of Manchester, Oxford Road, Manchester, M13 9PL UK; MRC Clinical Trials Unit at University College London, 125 Kingsway, London, WC2B 6NH UK; North West Hub for Trials Methodology Research, University of Liverpool, 1st floor Duncan Building, Daulby Street, Liverpool, L69 3GA UK; Health Services Research Unit, University of Aberdeen, Foresterhill, Aberdeen, AB25 2ZD UK; North West Hub for Trials Methodology Research and Department of Biostatistics, University of Liverpool, 1st floor Duncan Building, Daulby Street, Liverpool, L69 3GA UK; North West Hub for Trials Methodology Research and Department of Psychological Sciences, University of Liverpool, 1st floor Duncan Building, Daulby Street, Liverpool, L69 3GA UK

**Keywords:** Trials, Recruitment, Retention

## Abstract

**Background:**

Despite significant investment in infrastructure many trials continue to face challenges in recruitment and retention. We argue that insufficient focus has been placed on the development and testing of recruitment and retention interventions.

**Methods:**

In this current paper, we summarize existing reviews about interventions to improve recruitment and retention. We report survey data from Clinical Trials Units in the United Kingdom to indicate the range of interventions used by these units to encourage recruitment and retention. We present the views of participants in a recent workshop and a priority list of recruitment interventions for evaluation (determined by voting among workshop participants). We also discuss wider issues concerning the testing of recruitment interventions.

**Results:**

Methods used to encourage recruitment and retention were categorized as: patient contact, patient convenience, support for recruiters, monitoring and systems, incentives, design, resources, and human factors. Interventions felt to merit investigation by respondents fell into three categories: training site staff, communication with patients, and incentives.

**Conclusions:**

Significant resources continue to be invested into clinical trials and other high quality studies, but recruitment remains a significant challenge. Adoption of innovative methods to develop, test, and implement recruitment interventions are required.

**Electronic supplementary material:**

The online version of this article (doi:10.1186/1745-6215-15-399) contains supplementary material, which is available to authorized users.

## Background

There is currently a worldwide drive to enhance health, wellbeing, and wealth through effective research and dissemination. In the United Kingdom, the overarching vision of the National Institute for Health Research (NIHR) is to see ‘more patients and health professionals participating in health research’ [1].

A critical part of the health research portfolio is the testing of interventions through randomized controlled trials. Trials can range from highly controlled explanatory trials through to pragmatic trials of new health technologies and models of service delivery. A large number of trials are dependent on the willingness of patients and professionals to give their time and effort to participate. If high levels of participation (through recruitment to the study and longer-term retention) are not achieved, this has implications for statistical power, internal validity, and external validity. Recruitment problems also have practical and financial impacts, as they can delay completion of research or reduce its timely impact on patient health and wellbeing.

Achieving appropriate levels of patient and professional participation has been a significant obstacle to evidence-based practice. Published data show that the minority of trials recruit successfully, either in terms of reaching their planned sample size, or delivering the planned sample in the expected recruitment window [2, 3]. Although there may have been improvements since these initial surveys, related in part to the significant investment in infrastructure [4], problems still remain [5]. A recent survey of Clinical Trials Units (CTUs) in the United Kingdom conducted by the some of the authors [6] found that recruitment remained the number one priority of those units.

A recent review outlined three core areas of relevance in improving recruitment and retention: infrastructure (for example networks, resources, and information technology), professional and public engagement with research, and methodological innovation (the development of an evidence base around effective methods of recruitment) [7]. This current paper is designed to provide an overview of the current knowledge and practice in the area of methodological innovation, in order to set out a clear research agenda for the future.

### Methodological innovation

Many insights into the recruitment and retention process have been generated from qualitative case studies conducted alongside existing trials [8–11], as well as research on hypothetical situations [12, 13]. However, translating those insights into enduring and generalizable impacts on recruitment is not straightforward. Although this may be due to other limitations in the academic literature (such as the lack of robust theory to guide intervention development), the limited impact of this work may in part reflect the fact that these (essentially *post hoc*) explanations of recruitment processes are rarely subjected to formal examination in prospective studies. From the perspective of the principal investigator struggling with recruitment problems, this research has generated hypotheses to be tested rather than proven levers to ease recruitment.

The most robust test of the effectiveness of a recruitment or retention method is a trial comparing one recruitment method with an alternative, ‘nested’ within an ongoing trial being conducted in routine settings. By ‘nesting’, we refer to patients being randomly allocated to two or more alternative methods of recruitment. For example, a published study randomly allocated patients to an opt-in (where they were asked to actively signal willingness to participate in research) or opt-out method (where they were contacted repeatedly unless they stated unwillingness to participate) [14]. Such studies allow a less biased and more externally valid assessment of the effectiveness of a recruitment intervention. Nevertheless, despite the vast amount of activity in the area of clinical trials, nested studies of recruitment interventions remain very rare [15–17].

In this paper, we draw on a number of sources of data (including existing reviews on recruitment and retention interventions, survey data from CTUs in the United Kingdom, and views of participants in a recruitment workshop) to meet the following aims: to summarize knowledge about interventions to improve recruitment and retention, to indicate the range of interventions used by CTUs in the United Kingdom, to present a priority list of recruitment and retention interventions for evaluation, and to consider wider issues concerning the testing of recruitment interventions.

### Summary of current knowledge on recruitment and retention

Interventions to improve recruitment have been the focus of a number of systematic reviews. A Cochrane review collated randomized and quasi-randomized controlled trials of interventions to increase recruitment to trials, including those recruiting to hypothetical studies [15, 16]. The review included 45 trials involving 46 interventions and over 43,000 participants. Some interventions were effective in increasing recruitment, such as telephone reminders to non-respondents (risk ratio (RR) 1.66, 95% CI 1.03 to 2.46), use of opt-out rather than opt-in procedures for contacting potential participants (RR 1.39, 95% CI 1.06 to 1.84), and open designs whereby participants know which treatment they are receiving in the trial (RR 1.22, 95% CI 1.09 to 1.36). A substantial problem noted by the reviewers was the tendency for investigators to evaluate new interventions that are unlike earlier interventions, making pooling data difficult. This has resulted in a large pool of relatively unique recruitment interventions of uncertain benefit. Other reviews [18, 19] came to similar conclusions, although one review found no evidence that strategies aiming to increase understanding of the trial process improved recruitment, but did find some support for strategies that increased understanding of the health problem being studied [18].

Fletcher *et al*. [20] focused on strategies aimed at increasing the recruitment activity of clinicians and found eight quantitative studies, only three of which were trials. One trial looked at the effect of using nurses rather than surgeons to recruit participants and found that this had little or no effect (RR 0.94, 95% CI 0.76 to 1.17), though it was more cost-effective. There was limited evidence that greater communication between central trial coordinators and trial sites, and on-site monitoring had no impact on recruitment. The use of qualitative methods to identify and overcome barriers to clinician recruitment activity appeared promising, although the picture was mixed, with impressive improvements at one centre and no or modest improvements at others. The approach is certainly worthy of further investigation. A Cochrane review of incentives and disincentives to participation in trials by clinicians found no trials of relevant interventions [21]. The impact of a number of potential (dis)incentives was explored in observational studies but none were shown to have a significant impact. The authors suggested that in the absence of robust evidence, researchers need to be aware that many aspects of trial design and conduct might affect clinicians’ willingness to invite patients to participate.

In summary, there are some promising strategies for increasing recruitment to trials. However, some of those methods (such as open-trial designs and opt-out strategies), must be considered carefully as their use may also present methodological or ethical challenges. Use of qualitative methods to explicitly identify and address barriers to participation appears promising and warrants greater evaluation. There is a clear knowledge gap with regard to effective strategies aimed at recruiters.

Retention strategies have been the subject of three systematic reviews. Most of the retention strategies evaluated have focused on improving response to postal or electronic questionnaires, rather than return to trial sites to complete face-to-face assessments. A Cochrane systematic review on methods to increase response to postal and electronic questionnaires included 513 trials, with 137 strategies identified [22]. The most effective strategies to improve postal questionnaire response were: monetary incentives (odds ratio (OR) 1.87, 95% CI 1.73 to 2.04), recorded delivery (OR 1.76, 95% CI 1.43 to 2.18), a teaser on the envelope (OR 3.08, 95% CI 1.27 to 7.44) and having a more interesting questionnaire topic (OR 2.00, 95% CI 1.32 to 3.04). Other communication and questionnaire modification strategies found to be effective were: including pre-notification reminders, follow-up contact with participants, shorter questionnaires, and providing a second copy of a questionnaire. Several effective strategies for increasing responses to electronic questionnaires were found which included: including a picture in an email (OR 3.05, 95% CI 1.84 to 5.06), non-monetary incentives (OR 1.72, 95% CI 1.09 to 2.72) and other communication, and motivational and electronic questionnaire strategies. However, mentioning ‘Survey’ in the email subject line (OR 0.81, 95% CI 0.67 to 0.97), and emails including a male signature (OR 0.55, 95% CI 0.38 to 0.80) reduced the odds of a response. An earlier systematic review also focused on ways to increase the response to postal questionnaires in healthcare research [23]. A total of 15 trials were included in this review. Reminder letters (OR 3.7, 95% CI 2.30–5.97) and shorter questionnaires increased response (OR 1.4, 95% CI 1.19 to 1.54). Monetary incentives were not found to be effective.

These reviews were broad and included nested evaluations of strategies to improve retention in surveys, cohort studies, and randomized trials. Although some of the included trials in the reviews were nested in trials, most were nested in other study designs and the results may not be directly applicable to trials. A recent systematic review examined the effectiveness of strategies to improve retention in randomized trials specifically, and found 38 trials that evaluated six different types of strategies [17]. Most of the included trials aimed to improve questionnaire response. Questionnaire response was improved by actually adding monetary incentives (RR =1.18, 95% CI 1.09 to 1.28), the offer of monetary incentives (RR =1.25, 95% CI 1.14 to 1.38), and higher value incentives (RR =1.12, 95% CI 1.04 to 1.22). Based on results of single trials, response was improved by recorded delivery of questionnaires (RR =2.08, 95% CI 1.11 to 3.87), a specialized postal strategy (RR =1.43, 95% CI 1.22 to 1.67) and an open-trial design (RR =1.37, 95% CI 1.16 to 1.63). There is no clear evidence that questionnaire response or retention were improved by any of the other incentives, questionnaire modification, and communication strategies evaluated including the giving or offering gifts, offering charity donations, shorter or longer and clear questionnaires, sending questionnaires early, ‘enhanced’ letters (i.e. letters which contained additional information about trial processes or which included novel features, such as the signature of the main investigator), priority post, additional reminders, questionnaire question order, reminders to sites, and behavioral or case management strategies.

In summary, offering and giving small monetary incentives improves questionnaire response in randomized trials, while non-monetary incentives and some communication strategies have shown no effect. Some strategies need further evaluation, particularly where the results are based on single trials.

## Methods

In the United Kingdom, funding bodies increasingly require that trials involve a United Kingdom Clinical Research Collaboration registered CTU to ensure high quality delivery and appropriate support with ethical, governance, operational, and methodological issues. Due to their active involvement with multiple trials, CTU staff are potentially in an excellent position to provide an overview of current methods used to stimulate recruitment and retention.

In order to provide data on current practice, 48 CTU directors in the United Kingdom were sent an invitation to an online survey about the methods and practices currently used by CTUs to improve recruitment and retention. Directors were asked to identify a member of staff best placed to provide responses on behalf of the unit. Where more than one member of staff from the same CTU completed the survey, similar responses were combined to ensure that responses from the same CTU were not counted twice. Respondents were asked about the methods used to improve recruitment and retention (with or without formal evaluation), methods which had been formally evaluated, and recruitment and retention interventions thought to merit evaluation in the future. The full list of questions is provided in Additional file 1. Two reminder emails were distributed to encourage responses from all CTUs.

The results from the CTUs survey were used to inform a workshop on interventions to improve recruitment and retention, organized by the Medical Research Council North West Hub for Trials Methodology Research on behalf of the Medical Research Council Hub for Trials Methodology Research Network. Attendees at the workshop (n =45) were predominantly staff from CTUs (approximately 80%), as well as researchers outside CTUs, and representatives from funding agencies from the United Kingdom. Data from existing Cochrane reviews (summarized previously) were used alongside data from the survey to generate discussion around recruitment interventions. The final part of the workshop was used to generate further priorities for evaluation. Participants were split into small groups and asked to reflect on the data from the survey and the reviews, and to develop a priority list of interventions that would potentially improve recruitment and could be subjected to empirical testing. Groups reported back at the end of their discussions on both the nature of those interventions and their priority order, and the results were categorized by the workshop leader (PW). As the survey and workshop used professionals and involved discussions of current practice, no formal ethical approval or consent was deemed necessary.

## Results

Responses were received from 23 individuals representing 18 CTUs (38%). Respondents included statisticians, trials managers, health researchers, and research nurses.

### Current recruitment and retention interventions

Table 1 shows the methods routinely used to encourage recruitment and retention, which were coded into the following categories: patient contact, patient convenience, support for recruiters, monitoring and systems, incentives, design, resources, and human factors. These broadly map onto the categories of recruitment interventions found in the recent Cochrane review discussed previously [15].

**Table 1:** **Clinical Trials Unit survey on recruitment and retention - routinely used methods (with or without formal evaluation)**

Category	Examples (recruitment)	Examples (retention)
**Patient contact**	Patient information (appropriate design and translation)	Additional contacts (reminders, newsletters, feedback for patients, and websites)
	Promotion (newsletters, advertisements, presentations, events, press release, and community sessions)	
**Patient convenience**		Flexible appointments
		Reducing research burden (shortened assessment scales and online data collection)
**Support for recruiters**	Presentations and training about recruitment issues to recruiting staff	Presentations and training about retention issues to recruiting staff
**Monitoring and systems**	Recruitment staff reminders (computer pop ups)	Tracking patients (flagging, contacts for change of address, and collection of multiple contacts)
	Use of existing registers (mail shots and screening notes)	Reminders (calendars, alert cards, and ongoing contacts with control or wait list participants)
	Reducing burden (randomizing online in real-time, phone number for queries, and simple case report forms)	
**Incentives**	Targets (site recruitment targets, feedback, and competition among sites)	Incentives (gifts for sites, co-authorship for good retention, and monetary incentives)
	Incentives (gifts for sites, co-authorship for good recruiters, and monetary incentives)	
**Design**	Relevance of study design	Options other than complete withdrawal
	Piloting	Patient and public involvement
	Changing protocol (widening criteria)	
	Patient and public involvement	
**Resources**	Site resources	
	Additional resources (such as networks)	
**Human factors**	Relationships (face-to-face initiation visit, regular contact with recruitment staff, site champions, and ongoing relationships between trials)	Relationship (support for patient between visits, handwriting envelopes, Christmas/birthday cards, and thanking participants)

Patient contact interventions in recruitment related to appropriateness of the materials and the range of ways of getting information to patients, whereas retention was more focused on the number of contacts with patients. Both recruitment and retention interventions highlighted ways of reducing burden on patients, although it is not clear that research burden is necessarily the main barrier to participation. A large number of systems and monitoring interventions were discussed, to expand the range of methods used to identify patients, and to enable participants to be identified in the longer term as the trial progresses. Incentives included a wide range of potential interventions, such as direct payment for recruiters, patient expenses and gifts, and secondary incentives such as authorship on papers for staff involved in recruitment. Design issues were most often discussed in relation to recruitment, and included initial appropriateness of the design, the importance of pilot and feasibility studies, as well as flexibility in response to difficulties of recruitment. Respondents highlighted the importance of relationships in both recruitment (with the focus on relationships between the research team and recruitment staff) and retention (in terms of building and maintaining relationships with patients).

Table 2 describes interventions felt to merit investigation by respondents, in three categories: training site staff, communication with patients, and incentives. Some of these areas have been assessed in existing reviews, for example, site visits and intensive communication have been the subject of two studies with a published review, with little demonstrable effect on recruitment [20]. It is noteworthy that the impact of patient and public involvement was raised in two themes, given recent observational research suggesting an association between patient involvement and recruitment success [24]. Although the use of patient and public involvement is likely to be too embedded in current research to test its impact compared with an absence of involvement, exploring the relative benefits of different types of patient involvement, or different levels of resourcing of involvement is still likely to be of benefit to the research community.

**Table 2:** **Clinical Trials Unit survey on recruitment and retention - example methods thought to merit formal evaluation**

Category	Examples
Training site staff	Do site visits aid recruitment?
	What is the impact of site and investigator experience?
	What is the effectiveness of computer prompts?
	What is the comparative effectiveness of research nurses versus generic nurses?
	What is the impact of patient and public involvement?
Methods of communication with patients	What is the effect of publications and news releases?
	What is the comparative effectiveness of full information booklet versus a short invitation letter?
	What is the effect of telephone and/or text appointment reminders?
	What is the comparative cost efficacy of radio advertisements versus newspapers?
	What is the impact of patient and public involvement?
Incentives	Do small incentives (books or pens) improve recruitment rates more than acknowledgement in a newsletter or email?
	What is the comparative effectiveness of monetary incentives versus expenses?

### Priorities for evaluation - results from the workshop

The results from the CTUs survey were used to inform a workshop on interventions to improve recruitment and retention, using small group work to generate further priorities for evaluation. Table 3 details the results of the small group work. The top priority identified was training for site staff, followed by different methods of communication with patients. The following sections provide more information about the potential priorities within those areas that were generated at the workshop and through follow-up teleconferences among workshop participants.

**Table 3:** **Priorities for evaluation from the workshop**

Priority ranking	Intervention	Category
1	Training site staff and observation of recruitment	Training site staff
2	Use of different media to convey information, for example video, podcasts, and advertising	Methods of communication with patients
=3	Shifting information from the patient information leaflet to face-to-face discussion	
=3	Patient preference for information	
=3	Financial incentives for patients (need for public opinion on subject)	Incentives
=3	Incentives for site the and/or site staff	

### Training site staff

Many trials involve direct communication between patients and recruitment staff, and there is variability in the ability of staff on the same trial to achieve high levels of recruitment, with some studies reporting high levels of recruitment from a minority of practitioners [25]. This may reflect factors other than differences in their patient populations, such as variation among staff in the perceived importance of the study question, or different attitudes to equipoise. Identification of the characteristics of staff associated with recruitment and retention success could lead to a better selection of staff, while comparison of staff with different levels of recruitment success within the same trial might provide insights into effective components of training which could be developed into relevant training packages prior to formal evaluation. Such development will need to take into account the current debates concerning the ethics around coercive communication [26, 27]. There is also an interesting empirical question concerning the relationship between strategies that enhance recruitment, and effects on retention, as there is the possibility that encouraging ambivalent patients into studies may lead to short-term gains in recruitment, and longer term challenges in retention. The need to evaluate different models of verbal communication (for example empathic communication versus information provision) and to gain evidence of whether changes to recruiter communication behaviour leads to benefits for patients beyond recruitment rates (for example, improved satisfaction with the recruitment process and perceptions of shared decision-making) were also identified. Emphasis was placed on understanding patient priorities at the time of recruitment and how these may change over time to aid retention [28].

The relevant impact of generic communication skills versus specific skills around particular issues is an important issue. For example, discussions around patient preferences are known to be a major potential barrier to trial participation [29] and specific training in managing those discussions might be more fruitful than generic interventions, especially in certain contexts where preferences are particularly important [28]. However, studies continue to show problems in the core aspects of communication [30]. Another important issue is whether training should be provided at the start of any trial, for all recruiters, or whether it is more feasible and efficient to identify staff with low recruitment rates and intervene later, potentially following detailed qualitative work to identify the precise nature of the problems [31, 32].

### Methods of communication with patients

As noted, the focus of much of the discussion around training site staff was around the issue of face-to-face communication, whereas this theme related more to different types and platforms for communication with patients, and the balance between face-to-face discussions, other forms of providing information to patients [33], and wider interventions related to shared decision-making [34]. The use of technology for communication was highlighted in particular for recruitment in trials where the initial recruitment is not via a face-to-face consultation (such as community-based trials among patients with existing conditions recruited initially by postal or other methods). Technology was also considered to be an area that could assist with the retention of participants over time, both through effective tracking of patients and methods used to enhance motivation to continue participation (such as reminders and updates about trial progress).

Given the dissatisfaction among patients and staff over the potential length and burden associated with standard patient information sheets, technology would also potentially provide flexible and patient-centered methods to provide information in appropriate depth according to patient preference (as long as it meets minimum criteria as set by ethical and regulatory bodies) [35].

### Incentives

As noted in Tables 1 and 2, a wide range of potential interventions acting as incentives are in use and of interest to staff currently involved in the recruitment to trials, but the evidence base is limited [10, 21]. In relation to patients, this may include payment for time taken to participate (which might not be viewed formally as an incentive, although it might have motivational benefits), small gifts and payment for incidental expenses, as well as formal cash or voucher incentives for participation and retention. However, the scope for testing such incentives through formal experimental methods may be limited by ethical and equity considerations.

Issues of incentives can also be applied to professionals, although the scope here may be greater, as potential incentives could be indirect (such as authorship on papers). There may also be greater potential for experimental work in the testing of the comparative effectiveness of schemes which provide differential incentives for different recruitment staff, teams, or sites depending on their relative performance (per patient recruited incentives versus block payments for meeting targets).

### What is needed to facilitate rapid testing and development of interventions?

Although there was agreement about the need to conduct research on recruitment, the actual number of recruitment interventions nested within existing trials is very small [15]. Research has highlighted some of the known barriers to undertaking such research [36] such as increases in complexity, compatibility between the host trial where the recruitment research is done and nested trials (for example, the relevance of certain recruitment interventions to certain patient populations), the impact of nesting interventions on relationships with collaborators, as well as issues of preferences among research staff (and resulting lack of equipoise), and concerns about appropriate sample size.

Data on these issues were also collected from the CTUs survey, and the results generally fell into three categories. The first related to the logistics of running nested studies, in terms of the extra resources required, additional complications that might be caused to the delivery of the host trial (such as regulatory delays), and ethical barriers. The second barrier was a lack of perceived equipoise around many proposed recruitment processes and lack of enthusiasm in subjecting them to formal testing. The third category related to scientific issues, including concerns about the power to detect what might be quite small effects from methodological innovations, and the likely impact of variation in the effects of recruitment interventions, in terms of their effects on different sites, in different trials, and at different times.

## Discussion

### Limitations of the study

The CTUs survey was limited by the 38% response rate, and it is possible that non-responding units manage recruitment and retention differently from those included in the survey. Workshop participants (academics and staff in CTUs) represent key stakeholders, but the views of those attending a workshop on recruitment and retention may not have been representative of the wider trials community, and the findings would need confirmation in other contexts. Importantly, different priorities may be identified by other stakeholder groups, and in different countries. Particularly, there is a need to replicate these findings with patients and carers as core stakeholders in recruitment and retention. In this study, the CTUs survey was used to develop a list of recruitment interventions to feed into discussions in the workshop, but the content generated by the survey and the priorities generated by the workshop were not formally triangulated in any way.

### What are the limits to the impact of recruitment and retention interventions?

As noted previously, a recent review outlined three core areas of relevance to improving recruitment and retention: infrastructure, stakeholder engagement with research, and methodological innovation. In this paper, we have focused on methodological innovation, which we believe has an important part to play in improving recruitment and retention performance, and has the advantage being able to be evaluated and implemented throughout the platform of current clinical trials in a rigorous and controlled manner. Although the results are limited somewhat by the low response rate and the potential for bias, they do give a unique indication of the views of CTUs currently involved in recruitment and retention.

However, it is unclear how much variance in recruitment and retention performance is due to technical issues amenable to methodological research, compared to other issues such as available infrastructure, the organization, leadership, management and culture of research teams, and attitudes and values within the wider community. For example, staff in primary care networks in another workshop identified ‘positive attitudes of primary care staff towards research’ and ‘trust of researchers by potential participants’ as key contextual factors [37]. These factors are not necessarily those that are the most amenable to empirical testing, especially in a formal randomized comparison, although there are relevant examples [38].

It is noteworthy that many of the issues felt by our respondents to be worthy of evaluation are likely to have relatively modest effects on recruitment or retention, although this may reflect the fact that suggestions for interventions of higher impact (such as incentives) may be viewed as of low feasibility because of regulatory and ethical barriers. The scientific benefits of modest impacts on recruitment may be small (an increase in recruitment rates from 10 to 15% may have little substantive effect on external validity), although the benefits in terms of logistics, time taken to recruit, and trial funding may still be significant, given that the recruitment period may be a key driver of the length of a trial and its overall cost. Smaller benefits may be more important in retention.

It is possible that issues of efficiency are equally important. For example, rather than adopting methods with the aim of increasing the proportion of eligible patients who participate, studies may focus on whether more efficient methods can be adopted to maximize the number of patients who can be approached (although this is of course less relevant in certain contexts, such as rarer diseases). For example, in primary care, many trials have adopted postal recruitment using existing disease registers in preference to traditional recruitment led by clinicians [39]. The proportion of patients recruited by such postal methods may be equal to or less than traditional methods, but allows recruitment over a wider geographical area whilst using the same resources.

### A research agenda for recruitment and retention interventions

The results of this survey and workshop raise a number of implications for a future research agenda in this area. Experienced trial staff may have implicit ideas about what works in recruitment and retention, and a wide variety of factors are thought to be relevant. Some of these are likely to reflect good research practice and may not need or warrant empirical testing. However, given the importance of recruitment and the disruption it can cause, there is a surprisingly limited consensus on what needs to be tested to make recruitment practices more evidence-based. Authors of systematic reviews have commented that interventions that do get tested often bear an uncertain relationship to those in the broader literature, making pooled analyses difficult. We have highlighted three core areas that were felt to be a suitable focus for future work, and have considered some of the issues that might be amenable to testing. Further advances in this area may well be facilitated by the development and adoption of frameworks and typologies of recruitment methods of the type that have been adopted in other areas exploring complex, behavioral interventions [40]. This would involve describing categories of interventions and their potential mechanisms of effect, as well as potential moderating factors, such as the impact of different patient and trial characteristics. As well as providing benefits in terms of the development of effective interventions, this would allow more effective pooling of analyses at the synthesis stage.

Experienced trials personnel such as those involved in the surveys and workshops may be used to dealing with a lack of equipoise among clinical staff [41]. Therefore, it is noteworthy that there is not always equipoise among such staff about the effects of recruitment interventions, which can potentially act as a barrier to their evaluation. This raises the issue of how the delivery of nested recruitment interventions can be better incentivized. For example, individual trial teams and CTUs might receive additional resources to support their attempts to nest recruitment and retention studies in their trials to increase the adoption of this approach.

Scientific objections to evaluations of recruitment and retention interventions around issues such as power and heterogeneity are reasonable, although effective categorization, pooling, and meta-analysis could allow for the testing and consideration of many of these issues. The Medical Research Council Systematic Techniques for Assisting Recruitment to Trials (MRC START) program [42] and related initiatives such as Studies Within A Trial (SWAT) [43] and TrialsForge [44] may encourage a common framework across recruitment interventions and pooling to provide a more precise estimate of their effects, and to explore variation in their effects across patient populations, trial types, and recruitment contexts.

## Conclusions

Significant resources continue to be invested into clinical trials, but recruitment and retention continue to be problematic and remain high priorities among CTUs in the United Kingdom. There continues to be a major gap in the evidence base regarding what works in recruitment and retention. These findings provide guidance on areas that may be prioritized in the funding of further methodological research in this important area.

## Electronic supplementary material

Additional file 1: **Clinical trials unit survey questions on recruitment and retention.** (DOCX 15 KB)

## Abbreviations

95% CI: 95% confidence interval
CTU: Clinical Trials Unit
OR: Odds ratio, RR: risk ratio.
