# Supplementary material for: Interventions to improve recruitment and retention in clinical trials: a survey and workshop to assess current practice and future priorities
Source: Trials. 2014 Oct 16;15:399. doi: 10.1186/1745-6215-15-399 (PMC4210542; doi:10.1186/1745-6215-15-399)
Supplement: Supplementary file 1 — Additional file 1: Clinical trials unit survey questions on recruitment and retention. (DOCX 15 KB) [file 13063_2014_2265_MOESM1_ESM.docx]

**Additional file 1** Clinical trials unit survey questions on recruitment and retention

| Which methods/practices do you use routinely (with or without formal evaluation) to improve recruitment? |
| --- |
| Which methods/practices to improve recruitment have you evaluated? Please give as much as possible of the following details: situation prompting action, action/intervention  (and any comparator), method of evaluation, involvement of ethics committee, effect/results, impact on your work, publication details as appropriate. |
| In relation to improving recruitment, is there anything else that would influence your future practice which you have not described above? |
| Please outline any particular interventions in relation to recruitment that you think are of sufficient potential impact to merit formal evaluation. |
| What do you perceive to be barriers to the formal evaluation of recruitment interventions? |
| Which methods/practices do you use routinely (with or without formal evaluation) to improve retention? |
| For the methods/practices just described, what difficulties, if any, have you found with their implementation? |
| Which methods/practices to improve retention have you evaluated? Please give the following details: situation prompting action, action/intervention, method of informal evaluation, effect/results, publication details as appropriate. |
| In relation to improving retention, is there anything else that would influence your future practice which you have not described above? |
| Please outline any particular interventions in relation to retention that you think are of sufficient potential impact to merit formal evaluation. |
| What are the barriers to the formal evaluation of retention interventions? |
| Discipline(s) of person(s) completing survey |
| Experience that was drawn upon to complete this survey (at the level of the Clinical Trials Unit or the people who provided information to complete it) |
